# Severe Birth Asphyxia without Sequelae: A Case Report

**DOI:** 10.31729/jnma.4572

**Published:** 2019-08-31

**Authors:** Sujata Dahal, Roshan Lama, Nita Lohala, Prashant Simkhada, Meena Thapa, Sunil Raja Manandhar

**Affiliations:** 1 Kathmandu Medical College, Sinamangal, Kathmandu, Nepal; 2 Kathmandu University School of Medical Sciences, Dhulikhel, Kavrepalanchowk, Nepal; 3 Department of Obstetrics and Gynecology, Kathmandu Medical College, Sinamangal, Kathmandu, Nepal; 4 Department of Pediatrics, Kathmandu Medical College, Sinamangal, Kathmandu, Nepal

**Keywords:** *birth asphyxia*, *cord prolapse*, *hypoxic ischemic encephalopathy*

## Abstract

Perinatal asphyxia is one of the major causes of neonatal morbidity and mortality. It mainly causes neurodevelopmental delay leading to hypoxic-ischemic encephalopathy. We present here the case of a preterm male baby of 1670 grams born at 31^+3^ weeks of gestation delivered by 25-year-old Primi mother through vaginal delivery with history of umbilical cord prolapse. At birth, the baby had no heart rate and cyanosed following which he was resuscitated according to the Neonatal Advanced Life Support 2015 guidelines protocol. Even though after 5 mins of neonatal resuscitation, the baby's heart rate reappeared, but was only upto 20 beats/min and resuscitation thus continued. But heart rate did not improve despite of using all form of resuscitation procedure including intubation and drugs. But after 2 hours, baby cried spontaneously and later baby was managed in Neonatal Intensive Care Unit according to the neonatal unit protocol of the hospital.

## INTRODUCTION

Birth asphyxia occurs worldwide and remains a serious cause of mortality and morbidity of the newborn.^1^ Although most recent research has been focused on neurodevelopmental disability, the immediate issues that confront premature infants and their carers after birth are primarily related to systemic complications e.g respiratory distress, gastrointestinal dysfunction, with an increased risk of severe complications later such as Necrotizing Enterocolitis (NEC) and renal impairments including acute renal failure.^2^ It is interesting to note that this case of severe birth asphyxia even not responding initially with resuscitation, survived without neurological sequelae.

## CASE REPORT

A preterm male newborn of 1670 grams at 31^+3^ weeks of gestation was delivered vaginally with umbilical cord prolapse at Kathmandu Medical College Teaching Hospital, Sinamangal by 25-year-old Rh-negative primigravida with premature rupture of membrane. During intrapartum period, fetal heart sound (FHS) was less than 20 beats/min, for which patient party was counseled for the caesarean section but patient party denied. Thus, baby was born via vaginal delivery. The baby didn't cry after birth and was cyanosed with decreased muscle tone with no heart rate at birth, following which he was resuscitated by on duty

Pediatrics resident doctor. Apgar score at 1min, 5 min, 10 min and 15 min was 0/10, 1/10,1/10 and 1/10 respectively.

The baby was resuscitated as per Neonatal Advanced Life Support (NALS) guideline 2015 recommended by American Academy of Pediatrics (AAP).^3^ Even though heart rate appeared at 5 mins of birth after resuscitation, the baby was not breathing and there was no spontaneous respiration and heart rate was still below 20 beats/min. As heart rate did not improve despite using all form of resuscitation procedure including Bag and mask with chest compression, Intubation and drugs, treating doctors lost hope of surviving the baby. But after 2 hours, surprisingly baby cried spontaneously and later the baby was managed in the Neonatal Intensive Care Unit according to the neonatal unit protocol of the hospital.

**Figure 1.**
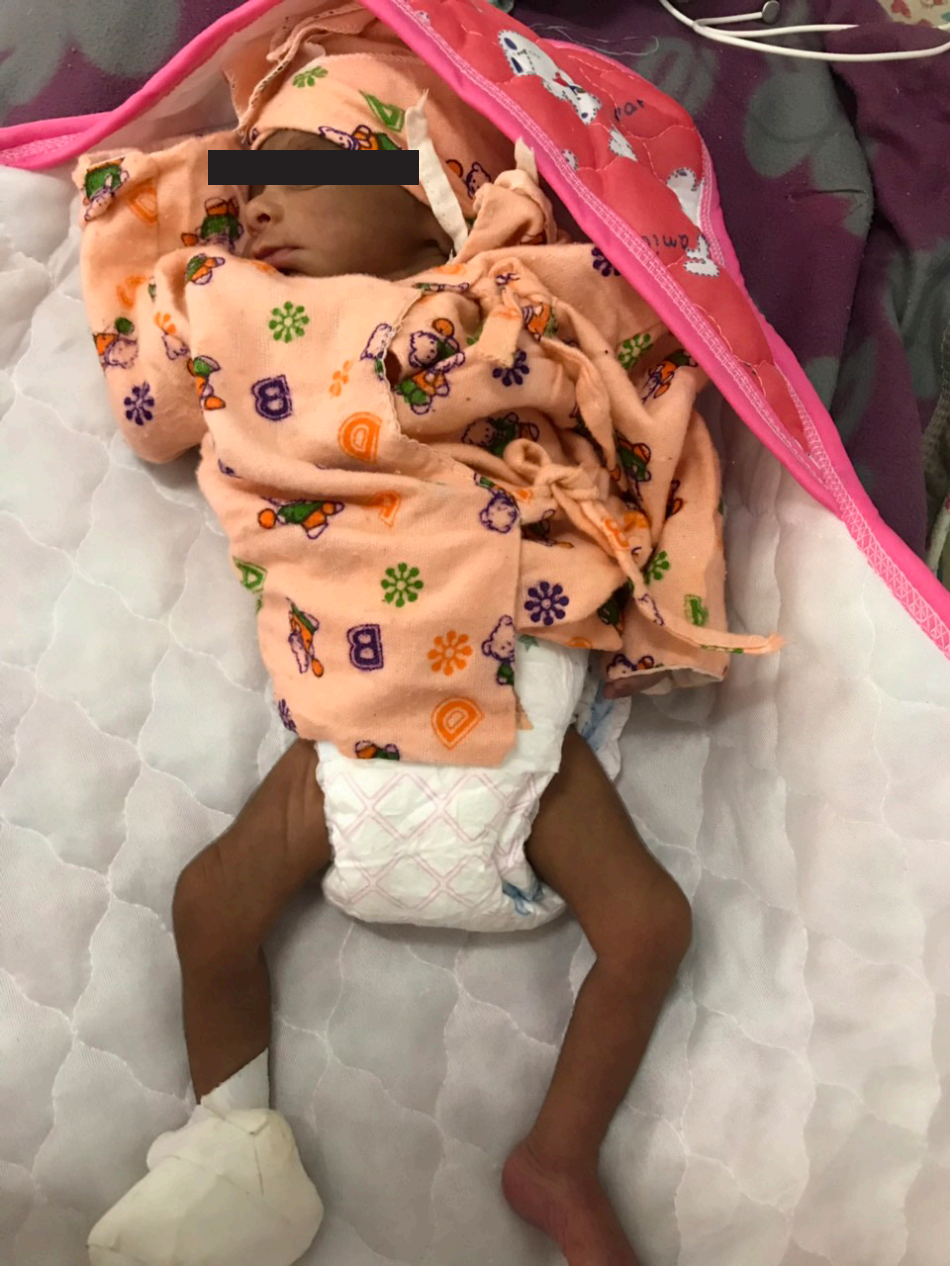
Preterm baby at day two of birth survived after severe asphyxia.

In Neonatal Intensive Care Unit (NICU), baby was kept under Mechanical ventilator CPAP (continuous positive airway pressure) with FiO2 50% for 24 hrs. In NICU, heart rate was within normal limit and SpO2 was 95%. On the 2^nd^ day of life, partial exchange was done due to polycythemia and baby was weaned off from CPAP and maintaining 98% saturation on room air without oxygen. The baby didn't develop seizure and blood sugar and calcium level and other routine blood investigations were also within normal limit. The baby was passing urine normally and the specific gravity of urine was 1.012. Feeding started on 3^rd^ day of life and the baby was tolerating EBM feeding (20ml/ 2hrly). Kangaroo Mother Care (KMC) was given for 2 days and the baby was discharged on the 7^th^ day of postnatal life from the hospital. The baby was followed up till one month of life and baby is normal with no neurological sequelae observed.

## DISCUSSION

The World Federation of Neurology Group defines asphyxia as a condition of impaired blood gas exchange leading, if it persists, to progressive hypoxemia and hypercapnia.^4^ In resource-rich countries, the incidence of severe perinatal asphyxia (causing death or severe neurological impairment) is about 1/1000 live births whereas, in resource-poor countries, perinatal asphyxia is much more common. Data from hospital-based studies in such settings suggest an incidence of 5–10/1000 live births.^5^

Perinatal asphyxia and birth injuries together contribute to almost 29% of these deaths.^6^ Failure to initiate and sustain breathing immediately after delivery has been associated with hypoxic-ischemic injury to the central nervous system and the clinical manifestations of this injury have been termed as Hypoxic Ischemic Encephalopathy (HIE). HIE is of concern in an asphyxiated neonate because it can lead to serious long-term neuromotor sequelae among survivors.^7^

For those who survive, outcomes have traditionally been divided into a dichotomy of impaired–non impaired: approximately 25% of infants show major neurological impairments, while the remaining 75% do not and thus are often classified as having a ‘normal’ outcome.^4^ It has long been recognized that its occurrence can be associated with brain damage and subsequent motor, cognitive and behavioral impairments.^4^

Antepartum or Intrapartum complications during labor also can also lead to HIE in babies. Cord compression may lead to an abrupt disturbance of oxygen supply with a sudden drop in fetal heart rate with persistent bradycardia. It is only possible to prevent hypoxic brain damage if the fetus can be delivered by emergency caesarian section within 15 – 20 minutes after the acute event.^8^ Mohanty reported a case of a late preterm neonate who died of severe perinatal asphyxia despite several attempts to intubate the baby.^9^

In a case report of Atiye Fedakâr severely asphyxiated newborn was also reported, male term baby weighing 2615 g with cesarean section due to fetal distress. His height was 46cm and the head circumference was 33cm. After birth, his overall condition was very poor, his heart was not beating, and he was not breathing. There was ecchymosis around the umbilicus and the chest area. He had respiratory depression and the Apgar score was 0 at the first minute and 2 at the fifth minute. The cornea was dull and pupillary light reflex was weak. The blood pressure was 70/30 mmHg. There was no other abnormal finding. Following resuscitation and intubation, he was referred to the intensive care unit with the diagnosis of severe asphyxia.^10^ In our case, male preterm newborn was delivered through vaginal delivery with cord prolapse. Despite having heart rate less than 20 /min after birth for a long duration (2 hours), the baby did not develop adverse outcomes of birth asphyxia i.e HIE and this remains beyond our understanding and scientific explanation.

Tertiary health care, availability of trained resuscitation team and availability of neonatal resuscitation facilities as recommended by the American Heart Association were available as supportive measures in this case for the revival of the baby. Long term follow up of the baby need to be done to make sure of the paucity of the adverse outcome later.
